# Adoption and implementation of produce prescription programs for under-resourced populations: clinic staff perspectives

**DOI:** 10.3389/fnut.2023.1221785

**Published:** 2023-10-27

**Authors:** Sara C. Folta, Zhongyu Li, Sean B. Cash, Kurt Hager, Fang Fang Zhang

**Affiliations:** Friedman School of Nutrition Science and Policy, Tufts University, Boston, MA, United States

**Keywords:** produce prescription programs, implementation, qualitative, clinic staff, food as medicine

## Abstract

**Background:**

Produce prescription programs represent a promising intervention strategy in the healthcare setting to address disparities in diet quality and diet-related chronic disease. The objective of this study was to understand adoption and implementation factors related to these programs that are common across contexts and those that are context-specific.

**Methods:**

In this qualitative case comparison study, we conducted qualitative interviews with eight clinic staff from five primary care “safety net” clinics, identified by a partnering non-profit organization that operated the programs, in April–July 2021.

**Results:**

Across clinics, the ability to provide a tangible benefit to patients was a key factor in adoption. Flexibility in integrating into clinic workflows was a facilitator of implementation. Fit with usual operations varied across clinics. Common challenges were the need for changes to the workflow and extra staff time. Clinic staff were skeptical about the sustainability of both the benefits to patients and the ability to continue the program at their clinics.

**Discussion:**

This study adds to a growing body of knowledge on the adoption and implementation of produce prescription programs. Future research will further this understanding, providing the evidence necessary to guide adopting clinics and to make informed policy decisions to best promote the growth and financial sustainability of these programs.

## 1. Introduction

Most U.S. adults have suboptimal diet quality despite some recent improvements, and disparities in diet quality have persisted or worsened for most dietary components (1, 2). These disparities in diet quality, in turn, contribute to disparities in chronic diseases (3). Healthcare systems can help address poor nutrition, and in this context produce prescription programs represent a promising intervention strategy.

Typically in produce prescription programs, patients with a diet-related health condition or food insecurity are referred by healthcare providers and receive economic incentives, often in the form of electronic cards or paper vouchers, to redeem for fruits and vegetables at retail food outlets (4). Retail food outlets may include grocery stores, farmers markets, or mobile markets. Programs often include some form of nutrition education, such as one-on-one counseling or classes, although the amount of nutrition education can vary considerably among programs (4, 5). While the effectiveness of these programs is still being studied, there is a growing body of evidence that they can positively impact fruit and vegetable consumption and food insecurity, as well as health outcomes including BMI, blood pressure, and glycated hemoglobin (HbA1c, or “A1c”) for patients in poor cardiometabolic health (6–8).

For these programs to achieve their potential in terms of public health impact, they will need to be widely and sustainably adopted within the healthcare system. It is therefore critical to understand factors that influence the decision to adopt these programs as well as barriers to and facilitators of their implementation and sustainability (9). It is also important to gauge provider perceptions of the impact of the program on patient outcomes. Qualitative methods are best suited to understand these implementation factors and perceived impacts because they allow for an in-depth examination of what is happening within clinics and why (10).

Few studies have examined factors related to the implementation of produce prescription programs. Most studies that examined implementation factors in-depth evaluated programs running at 3–4 clinics in a single state, and all partnered solely with farmers markets (11–13). These prior studies reveal several barriers, including staff time and turnover, different electronic health record (EHR) systems (11), and a lack of resources for sustainability (12); as well as facilitators: adapting clinic workflow (11), provider dedication (12, 13), and the ability to leverage existing relationships and programs (13). However, no consistent barriers or facilitators were revealed across these studies. To our knowledge, there is only one study in the peer-reviewed literature on implementation factors across multiple programs in different states. Stotz et al. (14) obtained perspectives from 16 health care providers from produce prescription programs that had been funded through the U.S. Department of Agriculture’s Gus Schumacher Nutrition Incentive Program (GusNIP). Common barriers included limited staff time and difficulty with patient and provider engagement; in terms of facilitators, EHR-based screening systems and full-time paid staff members to manage the program facilitated implementation in the clinics that had them.

While prior research identifies a number of barriers and facilitators to implementation, it does not fully address factors that may be common across contexts and those that may be context-specific. Furthermore, only one other study examines factors that influence clinics to adopt a program (11). We used a qualitative comparative case study approach to obtain perspectives from administrative clinic staff on produce prescription programs implemented in five “safety-net” clinics serving low-income populations that otherwise varied by geography, some aspects of the program, and populations served by the programs. This research contributes to and extends prior research to understand the factors related to adoption and implementation of produce prescription programs. This understanding will support the successful growth of these programs, since it can inform implementation at multiple levels: individual clinics, healthcare systems, and policy.

## 2. Methods

### 2.1. Design, setting, and sample

Our academic team partnered with a non-profit organization to conduct an evaluation of their produce prescription programs, including this qualitative assessment of factors related to adoption and implementation. The non-profit partner is a leading national organization that has supported and developed nutrition incentive programs throughout the U.S. The non-profit partner operated the produce prescription programs by recruiting clinics and retailers as well as securing funding for the programs. Funding sources included a state department of public health, a food company, and healthcare organizations. All clinics served low-income populations. In all programs, enrolled participants received a financial incentive (paper voucher or electronic gift card), delivered once per month, to purchase produce at grocery stores. The programs otherwise varied by geography, monthly dollar amount, nutrition education component, size of the program, and the specific population served (Table 1). The programs also varied in length: the shortest programs in this study were 6 months and the longest were 10 months (Table 1). Program duration varied based on the resources the non-profit partner was able to secure and by the goals of the partnering clinics. The variability among programs allowed us to take a qualitative comparative case study approach (15).

**Table 1:** Clinic characteristics.

Program	Number and title of staff interviewed	Type of program	Type of clinic	Eligibility	Geographic region	Dollar value of monthly produce prescription incentive	Program duration (months)	Program size (# participants)	Delivery mode	Nutrition education component
Clinic 1	1, Wellness coordinator and coach	Pediatric	Community health center	Children ages 1–5 years	South	$60	8	Large (>300)	Card	Two videos, approx. 60 min each
Clinic 2	1, Chief executive officer	Pediatric	Community health center	Children age 2–18 with household food insecurity	Northwest	$60	6–7	Medium (100–300)	Card	None
Clinic 3	1, Ambulatory care pharmacist	Adult	Community health center	Adults with diabetes or pre-diabetes.	Midwest	Year 1: $30 per household member; Year 2: $90 for all households	10	Small (<100)	Voucher	Group-based administered by RD
Clinic 4	1, Program manager	Adult	Community health center	Adults with diabetes or pre-diabetes.	Midwest	$90	10	Medium (100–300)	Voucher	Year 1: one-on-one counseling with clinic staff
										Year 2, online groups with RD
Clinic 5	4, Nurse manager, head of clinics, medical administrative assistant, business systems associate	Adult	Hospital	HbA1c > 6.5%.	Northeast	$60	6	Large (>300)	Voucher	Minimal; opportunity to connect with programming by RD

We interviewed staff from a convenience sample of five clinics that had partnered with the non-profit partner to implement a produce prescription program within the past 2 years. Interviews were conducted in April–July 2021. Clinic staff to be interviewed were identified by our partnering non-profit organization as the primary implementer at the respective clinics.

The research team has expertise in qualitative methods and implementation science (SCF), economics (SBC), and nutritional epidemiology (FFZ). The team also included a project administrator (ZL) and a doctoral student whose dissertation research focused on food is medicine interventions (KH). The team was concurrently working on a quantitative analysis of the produce prescription programs.

### 2.2. Interview procedures

The interview guide was informed by Proctor’s framework (9) and covered three areas: (1) factors related to the decision to adopt; (2) “implementation and logistics,” covering items such as fit with usual practices; and (3) “impact and sustainability,” including perceptions of the impact on patient outcomes and sustainability at the patient and clinic levels. A phenomenological approach was taken and the guide was semi-structured with open-ended questions. Table 2 contains the interview questions. Questions were developed by the team’s qualitative expert (SCF) with input from the rest of the research team, the non-profit partner, and a clinic staff member who had implemented one of the programs.

**Table 2:** Produce prescription program—interview guide for clinicians.

**Questions about adoption** Think back to when you were deciding whether to do this program. What motivated you to participate in the program? Tell me what you thought about the materials the clinic received from [non-profit partner]. What other information would you have wanted that you did not have when making the decision? Describe how easy or hard it was for staff to understand the program. Talk about any pushback there was from your administration about running the program at your clinic.
**Questions about implementation and logistics** Describe how well this program fit within your clinic’s usual practices. What was required to run the program in terms of personnel? Who did it fall to the most? Describe how easy or hard it was for staff to administer the program. Approximately how much “extra” time was spent by staff members with each program participant, compared to how much time they would spend during a visit when the program was not taking place? [Probes: nutrition education, registering patients, and follow-up with program patients]. To what degree was the time spent by any staff explicitly compensated by [non-profit partner]? [If informant does not know:] Who else on staff could better answer this? How involved were the doctors in the program? In your opinion, how much should doctors be involved with the implementation of the program vs. other clinic staff? How could the program be made more efficient for clinic operations? What costs, if any, were incurred by the clinic to run the program that were not covered by [non-profit partner]? [If informant does not know:] Who else on staff could better answer this? If you were to do this program over again in your clinic, what would you do differently? Describe how well this program fit within your own usual set of practices. In what ways, if any, did it become part of your routine? How adequate was the amount of time you had to manage the program? How adequate was the time you had to talk with patients about healthy foods? In what ways, if any, did the program affect the way you worked with patients? In what ways, if any, have those changes remained since the program ended? How did you recruit families for the program? What was most successful in terms of recruitment? What did not work as well? Describe how easy or hard it was patients to understand the program. What questions did patients ask about the program? What are your impressions of the adequacy of the produce incentive that the participants received from [non-profit partner]? What would have been the right amount?
**Questions about impact and sustainability** Talk about your impressions of the program overall. As you reflect on the program overall, in what ways did it meet your expectations? In what ways, if any, did it fail to meet your expectations? What, if anything, surprised you about the program? Tell me your thoughts about the program’s ability to help your patients – please do not mention any names or specific identifying information about these patients. In what ways, if any, was it a useful tool for them? In what ways, if any, did the program improve the quality of care you felt you could provide? What types of patients did it seem to work best with? Describe an example of a patient who had a very positive experience with the program. What made it such a great experience for that family? What types of patients did it seem to not work as well for? Describe an example of a patient who the program did not work for. Tell me your thoughts about whether your patients will sustain any changes in fruit and vegetable consumptions that they made during the program. What could the program do to help patients sustain the benefits? What could the clinic do? What would clinics need to be able to sustain the program?

Interviews were conducted online via the Zoom videoconferencing application either by SCF, ZL, KH, or by two graduate research assistants. SCF trained the other interviewers. All but one of the interviews were conducted one-on-one with the study team member and clinic staff. At Clinic 5, one of the interviews was conducted with both the medical administrative assistant and the business systems associate together. Interviews lasted 24–58 min. Participants received a $50 gift card for remuneration. The protocol was reviewed and deemed exempt by the Tufts University Health Sciences Institutional Review Board. Informed consent was obtained verbally from all participants.

### 2.3. Analysis

The interviews were recorded and transcribed, and then coded using NVivo software (version 12, QSR International, Doncaster, Australia). SCF and ZL were primarily responsible for the analysis. We used a directed qualitative content analysis approach, which is fundamentally deductive (16). We drafted an initial codebook based on the interview guide. We then conducted a review of the transcripts and added codes for topics that arose in the data. Once the codebook was established, SCF and ZL independently coded one transcript. We determined inter-rater reliability, with kappa coefficient of 0.8 or greater at each code deemed as acceptable. Based on this testing, we discovered minor differences in interpretation of codes; the codebook was revised accordingly, mainly by clarifying code definitions, and all transcripts were subsequently coded by ZL. The codebook remained stable at this point, reflecting code saturation (17). We then examined the data for both common themes and differences across the sites (15).

## 3. Results

We interviewed eight clinic staff from the five clinics in our sample. For clinics 1 through 4, we interviewed one staff member, who was the primary implementer. In clinic 5, because we had a strong research collaboration with the clinic, we were able to interview four clinic staff to obtain perspectives from multiple types of stakeholders. At that clinic, in addition to the primary implementer, we also interviewed the head of clinics, a medical administrative assistant, and the business systems associate. The multiple perspectives at this one clinic offered a well-rounded view, which enhanced but did not fundamentally change the results. While six clinics had implemented a produce prescription program in partnership with the non-profit partnering organization in the timeframe, one primary implementer failed to complete an interview due to leaving her position shortly after being identified.

We present findings according to major topics covered in the interview guide: adoption, implementation, and impact on patients and sustainability.

### 3.1. Adoption

Clinic staff at all clinics described enthusiasm about the program and a willingness to adopt it at all levels of decision-making (*…it seems like hierarchy-wise it was a support at all levels…—Head of Clinics, Clinic 5*). For willingness to adopt, there were similarities across sites that converged into two main themes: simplicity of the program and potential benefits for patients.

Clinic staff described the produce prescription program, as presented to them by the non-profit partner, as easy to understand. They said that staff at the non-profit partner had provided adequate information about the produce prescription program and had been available to answer any questions. Clinic staff perceived the produce prescription program as reasonably flexible and relatively free of “red tape” in comparison to other programs their clinics had adopted to help their patients (which were not produce prescription programs, and often not focused on nutrition specifically).

Clinic personnel were motivated to adopt the program because of the potential benefit to the patients, especially given the need in the populations that they served.

*So yeah, I'm dealing with an underserved population, whose—my mission is to support them, so that was a no-brainer. And many of our initiatives, again, like I said, come from partners from outside. So any partner outside willing to partner with us, with our patients, we tend to work on them with open arms, for the most part. (Head of Clinics, Clinic 5)*

*That was something our dietitian was hearing left and right, was that just fruits and vegetables cost too much. And so she's trying to work with them on changing eating habits and they're saying I don't have the access to those things, so we thought it was a great way of removing that barrier and exposing people to some of the benefits of fruits and vegetables. (Ambulatory Care Pharmacist, Clinic 3)*

Only one site described any pushback to program adoption. At Clinic 1, the primary implementer was enthusiastic about the program, but was initially concerned that the clinic did not have the patient base to meet the recruitment expectations of the non-profit partner. This concern was alleviated by opening the program up to community members who were not patients at the clinic.

### 3.2. Implementation

Because of the flexibility allowed by the non-profit partner, there was variation in how the program was implemented at each site and in the clinics’ experiences with the implementation.

*But [the non-profit partner] tried to stay pretty hands off, from the perspective of letting us develop something that was, you know, better for our own clinic, right. So they gave some guidance and they gave some ideas, but it's not like they handed us kind of like a turnkey program and just said go. They let us really develop what was—what we felt like was most needed for our patients and what worked within our workflow much, much better. (Ambulatory Care Pharmacist, Clinic 3)*

Interviewees described five major tasks involved with program implementation: (1) recruiting/identifying patients and determining eligibility; (2) distributing and tracking the produce prescription incentives; (3) responding to patient inquiries related to the incentive; (4) obtaining and tracking outcome measurements (which included participant fruit and vegetable intake, food insecurity, HbA1c, blood pressure, and BMI/BMI z-score; in some cases it required scheduling extra appointments for these measurements); and (5) providing nutrition education. These tasks fell to different staff members at the different clinics and for some, shifted over time.

Physicians were minimally involved in implementation at all clinics. Their involvement ranged from attendance at several community recruitment events to initial identification of eligible program participants. The various tasks related to the program fell instead to front office staff, nurses, medical assistants, registered dietitians, the pharmacist, a health and wellness educator, and a volunteer.

There was variation and no major theme identified around perceived fit with regular clinic operations. At Clinic 1, implementation was described as essentially a seamless fit with normal operations. At this clinic, a full-time health and wellness education professional was primarily responsible for implementing this and other special projects. In contrast, interviewees at the remaining clinics described challenges to implementation since the program required changes in standard workflows and put demands on staff time.

The interviewee at Clinic 2 described putting in upfront effort to integrate the program into the EHR and other systems. At this clinic, physician involvement initially presented a challenge, and they shifted implementation to front office and nursing staff. With the upfront effort to create the protocols and the change in implementer, the program fit well within usual clinic practices thereafter.

*And so, at first we were running those through the provider and some of the nursing team. But that became a little problematic, in that they would forget or, you know, [the patients are] there for diabetes and hypertension and some of these other things, and so the provider’s thinking about prescriptions and those kinds of things and they're not thinking about the gift card as well, so we quickly removed it from the provider piece and put it on the front office and the nursing. And so between those two, the nurses would recommend based on what happened in the office, and then the front office could verify that they didn't have, you know, that they were kind of an underserved patient…So they implemented—once we moved to that piece it went pretty smoothly. (Chief Executive Officer, Clinic 2)*

At Clinics 3 and 4, the interviewees described major challenges in the first year of the program. Both sites included a substantial nutrition education component that required adaptations in the second year due to the COVID-19 pandemic. In both cases, they found that the modified model, which was remote, group-based nutrition education implemented by a registered dietitian, worked better.

*It was interesting that the pandemic provided us the opportunity to do something that worked really well. I think that that support group type setting was something that provided a lot of efficiency. Working with registered dietitians as opposed to staff members who are involved in direct care and had a lot of patient appointments anyways, this seemed to more naturally fit into the registered dieticians' workflow. They were able to handle a lot of the follow-up that typically would have been—or in the previous year, was very burdensome to the staff that was involved. Obviously, there's always room for improvement, but that second year was really a good model of what worked well and would be something that we would probably mimic if we were going to be doing that for another year. (Program Manager, Clinic 4)*

Clinic 5 experienced the most serious challenges to implementation. Clinic staff perceived the program as time- and resource-intensive. Here, the four staff interviewed described the program as a “collective responsibility” that required thoughtfulness and effective communication among many members of the clinic staff. For part of the program, they had a volunteer for 20 h per week, and many responsibilities fell to this person.

*…we also had a volunteer at the time from a program that we work with at the hospital that filled a lot of the data collection and updating spreadsheets and things like that, so we were very lucky to have someone that didn't have their daily tasks, that were being pulled from like their daily responsibilities, and so she kind of took on the little coordinator kind of role…. (Nurse Manager, Clinic 5)*

In this busy clinic, even with minimal nutrition education, implementation required multiple tasks and remained challenging.

*…so the only thing was like because we have to do it like during—when the patient was here. And usually we do have a workflow, like when a patient arrives, do the vital signs so—and to incorporate the [outcome measures requested by the non-profit organization], so we have to change the workflow to see how that we can place the order for the hemoglobin A1c and then how we can communicate a provider to place the order, and then after that we have to do—do the whole information on the tablet with that ID number and things like that, so it was a lot of—(Business Systems Associate, Clinic 5)*

*—Lots of, yeah, lots of hands on—a lot of manual tracking, and yeah. (Medical Administrative Assistant, Clinic 5)*

### 3.3. Impact on patients and sustainability

There was a high level of similarity across clinics in how interviewees described the impact on patients. A major theme was that they appreciated having something concrete to give to patients to help their social, nutritional, and economic struggles and improve their health.

*…again it was another component in supporting the social determinants infrastructure by dealing with their basic needs of eating and eat—and hopefully eating better with the fresh fruits and vegetables. (Head of Clinics, Clinic 5)*

*So we've always kind of tried to help in some of those social determinants and some of those areas where our patients really struggle, but we've never been able to do it in a focus on being healthy as well, so that's where this really was an amazing benefit, is because now it was truly something that was specifically for people who we knew needed to be healthier and couldn't afford to do that. (Chief Executive Officer, Clinic 2)*

*I think anytime that we have something that we can actually give them, that's concrete ways to change their eating behaviors is really helpful. Rather than just telling people to eat better, we can give them something to help them eat better. (Program Manager, Clinic 4)*

Additional benefits that were mentioned by some of the interviewees, but not consistently across clinics, included patients feeling supported by the clinic, benefiting from the nutrition education, and increasing engagement with the clinic, especially during the pandemic.

*I think the interaction too with the staff was nice for the patients because even during Covid it was like people were isolated and you still had the staff reaching out to say hey, don't forget your vouchers or hey, another mailing is coming in…there was still communication going back and forth, even during Covid when patients might have felt like isolated. So yeah, I think again the benefit outweighed the amount of work it took to implement it. (Nurse Manager, Clinic 5)*

Most clinic staff thought that patients were satisfied with the monthly produce prescription incentive amount, even though the dollar amount varied somewhat across clinics.

A second theme related to impact on patients was that clinicians were skeptical about patients sustaining any changes that they had made without an ongoing incentive for free fruits and vegetables. Staff at clinics that had not provided much nutrition education felt that providing more in combination with incentives might be helpful in sustaining long-term dietary changes. Overall, though, the level of poverty among patients participating in the program was the basis for clinic staff skepticism of long-term program benefits. Staff expressed concern that without the additional dollars provided by the program, patients would have less ability to purchase the same amount of fruits and vegetables. The program manager at Clinic 4 suggested that financial education could possibly help the patients learn to fit produce into their budgets, an approach that had been tried at Clinic 3 in a small way at one of the final classes.

*I don't know if there is an amount of financial education or maybe working to get people other resources to show them how to do this. I think that maybe it's the potential of trying to find other ways to show patients or get them to acknowledge how improving their A1c made them feel, to the point where that would cause them to readjust some of their budget to include more fresh fruits and vegetables. Like I was saying before, just from some of the feedback, some people just have such deep poverty that that really is challenging. (Program Manager, Clinic 4)*

Only the wellness coordinator and coach at Clinic 1 was hopeful about the sustainability of changes. In this pediatric program, he felt that once parents saw that their children were willing to eat different types of produce, they would be more willing to purchase it after the conclusion of the program.

*And in that way, I think a lot of parents and participants were able to benefit from it, because they were able to experiment. So I think it really played a huge role, because even though they got off the program [because the program ended] they were using that $60 for six months, and they were able to say, okay, these were some of the things that work. And now they could—when they're investing their own money they can feel more confident knowing if I buy these grapes, my daughter's gonna eat them. (Wellness Coordinator and Coach, Clinic 1)*

In terms of sustainability of the program at the clinic level, there was consistency across clinics and a major theme was that the clinics would run the program again if given the opportunity, but they could not do so without external funding.

*And then funding, if you had a state that was willing to put some money into it to make it sustainable, or if you had a large, generous foundation or something like that that was willing to year over year really invest in to see, does this actually make a change over the course of, you know, 5 years of providing fresh fruits and vegetables to a family. And then see what the data would show, but without that kind of commitment, just like here, it was awesome while we had it. We’d definitely do it again in a heartbeat. But as soon as it went away, we look to the next kind of program to help our patients. (Chief Executive Officer, Clinic 2)*

*And there might be push back [if I asked the healthcare corporation for money for the program] like I do not—I do not have enough leverage, I think, with my population to extol resources from the program from the corporation. However, for, let us say a department that brings in revenue to the system. Let us say, like the bariatric surgery program. They may exert leverage to say, “I need additional resources to administer this program.” See my point, they—whereas mine I go into the CEO or I have poor patients who I do not get money for, can you give me resources to administer this program. As opposed to, “I’m bariatric surgery and bringing in X millions, and this would be a great adjunct to what we do.” Right, they have greater leverage. (Head of Clinics, Clinic 5)*

## 4. Discussion

This study is one of the first to explore implementation factors related to produce prescription programs across multiple program types. In this qualitative study, clinic staff described simplicity, flexibility and the ability to provide something tangible to their patients as factors in the decision to adopt the program. There were challenges to implementation at most clinics, and the need for changes to the workflow and extra staff time were common ones. Physicians were minimally involved compared to other clinic staff. Clinic staff expressed skepticism about the sustainability of both the benefits to patients and the ability to continue to offer the program at their clinics.

### 4.1. Adoption

Participants described several key factors related to adoption. A major factor in program adoption was compatibility, or the degree to which the innovation fit within existing values, experiences, and systems (18). While there were otherwise several major differences across the sites, all were “safety net” clinics serving very low-income populations, and staff indicated that their clinics were serial adopters of programming to help meet the needs of their patients. The produce prescription program was compatible with this practice and the flexibility also made it seem compatible with existing workflows. Perhaps also because of their prior experience with adopting other programs, clinic staff perceived a low barrier to entry, since the non-profit partner addressed most of the program cost. Clinic staff also described the program as being easy to understand, a factor related to the communications and materials provided by the non-profit partner. A theme in our study and in Stotz et al.’s (14) interviews with providers participating in GusNIP-funded programs was that clinic staff appreciated having something tangible to offer patients, which provided a relative advantage over other clinic-based programming. In another study of produce prescription programs connected to Medicaid in one state, a theme among multiple types of stakeholders, including healthcare payers, administrators, and researchers, as well as clinicians was that produce prescription programs likely represented a relative advantage in terms of increased patient engagement and satisfaction with healthcare (19). These characteristics may be useful in promoting further growth of produce prescription programs.

### 4.2. Implementation

In addition to being a factor in the decision to adopt, across the sites in our study, flexibility was also described as a key facilitator of implementation. Other studies conducted have consistently described the need for flexibility and adaptability in the implementation of produce prescription programs (11, 12, 20). However, in interviews conducted for a report by the Center for Health Law and Policy Innovation, some interviewees also indicated the need for standardized solutions to facilitate implementation and promote sustainability (20). In their implementation study of a program for hypertensive patients, some elements were standardized by providing clinic staff with training on how to counsel patients, develop a referral process, and integrate the program into the EHR system (11). This suggests that while flexibility is critical, some training and standardization may help ease the start-up issues that several of the clinics in our study described. It may also help increase adoption of produce prescription programs (21). Standardization could facilitate future comparisons across clinics, with the potential for improving the effectiveness of programs as the evidence starts to accumulate regarding best practices. Additional systematic studies of implementation will help clarify which aspects of the programs might be standardized and which should be left to the clinics to adapt to best fit with their local workflows.

While we found that flexibility was a common facilitator of implementation, staff time was a common barrier across the clinics. A solution to staff time issues suggested by our results is assisting clinics with upfront changes that will alleviate workflow inefficiencies, such as adding fields to the EHR. Noting the differences among the clinics, our results also suggest that staff time issues might be alleviated if responsibilities for managing the program fall to staff whose job is well-aligned, such as health educators. Similarly, in Stotz et al.’s (14) interviews with providers participating in GusNIP-funded programs, hiring a full-time staff member to manage all aspects of the program emerged in a theme on solutions to challenges. While a volunteer effectively served this manager role for Clinic 5, relying on volunteers is not likely to be a desirable model for most clinics. At Clinic 5, the volunteer’s time ended before the program did, causing further issues; also, Stotz et al. (14) address the inequitable nature of this unpaid labor.

Our results indicate that produce prescription programs can be implemented, potentially more effectively, by clinic staff other than physicians. The study with hypertensive patients had similar findings (11). Both that study and ours involved “safety net” clinics, in which physicians face greater time and other resource constraints in working with medically underserved populations (22). It will be important to continue to conduct implementation research to understand the best role for physicians and other clinic staff in different types of clinics.

### 4.3. Impact on patients and sustainability

While a major theme was that providing a tangible form of support had a positive impact on patients, all clinic staff expressed skepticism about sustainability at both the level of patients’ individual habits and at the clinic level. In a field scan of produce prescription programs, self-supported programs (funded through the organizational budget) lasted the longest, an average of 4.5 years, but only 4% were self-supported (4). Because of this issue, there is growing interest in policy solutions, such as coverage options within Medicare and Medicaid and the expansion of GusNIP programs (4, 20). In September 2022, the Biden administration released the National Strategy on Hunger, Nutrition and Health, which listed as priorities expanded Medicare and Medicaid coverage of produce prescriptions, universal screening for food insecurity in federal health systems, and produce prescription pilots in Indian Health Services and Veterans Health Administration (23). These policy developments signal a move toward greater sustainability and an increased need for effective and efficient implementation of produce prescription programs across a range of settings in the U.S. Yet, concerns about long-term sustainability have been raised by some produce prescription providers, given recent guidance from the Centers for Medicare and Medicaid Services that coverage of produce prescription programs should be limited to a maximum of 6 months (24).

### 4.4. Strengths and limitations

This study had multiple strengths and limitations. It is one of the first studies to compare adoption and implementation factors across clinic sites. However, the programs were all associated with the non-profit organization that partnered with our research team and therefore this study only includes perceptions related to the specifics of produce prescription programs associated with that organization. Another limitation is the small number of clinics in the sample. While the overall sample was small, it allowed us to compare across clinics. We also achieved code saturation (17), suggesting the robustness of themes. However, we may not have fully achieved data saturation in this small sample (25). Future studies that include a larger number and greater diversity of clinics will more fully address the similarities and differences in factors related to adoption and implementation of produce prescription programs.

### 4.5. Implications for practice

Based on our findings, the following points may be helpful to clinics considering the adoption of a produce prescription program:

- Communicating how the program fits with the values and practices of the clinic will help ensure that staff will persist in resolving barriers to implementation. The ability to provide a tangible support to patients was also a motivator for clinic staff.
- Ensuring that program logistics are simple and flexible may facilitate adoption and implementation.
- It is likely that up-front effort to build the program into workflows, including integrating it into the EHR system and possibly other forms of patient tracking, will pay off in terms of smooth implementation.
- Clinics may consider the involvement of multiple clinic staff to implement programs. Reliance primarily on physicians may impede implementation in some clinics.
- More intensive and targeted nutrition education and counseling (e.g., group classes, ideally led by a registered dietitian) should be considered in future programming. Especially in areas of high poverty, some form of financial education could be considered as well.
- Clinics may be unable to bear the cost of sustaining the program. Other solutions should be explored to ensure the sustainability of the program.

### 4.6. Conclusion

This study contributes information on factors related to adoption of produce prescription programs and corroborates previous research about barriers to implementation, including staff time. It also provides further evidence of common facilitators, such as the flexibility and adaptability of the programs. Our findings suggest the importance of policy solutions to ensure sustainability of produce prescription programs. This study described implementation in “safety net” clinics of programs that received resources from the same non-profit partner. Additional research is needed to understand implementation of produce prescription programs more fully across a range of settings, to further determine which barriers and facilitators are common and which are unique in different contexts. These studies will provide the evidence necessary to guide adopting clinics and to make informed policy decisions to best promote the growth and sustainability of these programs.

## Data availability statement

The original contributions presented in the study are included in the article/supplementary material, further inquiries can be directed to the corresponding author.

## Ethics statement

The studies involving humans were approved by Tufts University Health Sciences Institution Review Board. The studies were conducted in accordance with the local legislation and institutional requirements. Written informed consent for participation was not required from the participants or the participants' legal guardians/next of kin because the protocol was deemed exempt. Verbal consent was obtained.

## Author contributions

SF, SC, and FZ contributed to study conception. SF, ZL, and KH made substantial contributions to data acquisition. SF and ZL analyzed the data. All authors contributed to the article and approved the submitted version.
